# Enhanced dimension-specific visual working memory in grapheme–color synesthesia^☆^

**DOI:** 10.1016/j.cognition.2013.06.009

**Published:** 2013-10

**Authors:** Devin Blair Terhune, Olga Anna Wudarczyk, Priya Kochuparampil, Roi Cohen Kadosh

**Affiliations:** Department of Experimental Psychology, University of Oxford, UK

**Keywords:** Color-processing, *n*-Back, Grapheme-processing, Synesthesia, Visual, Working memory

## Abstract

There is emerging evidence that the encoding of visual information and the maintenance of this information in a temporarily accessible state in working memory rely on the same neural mechanisms. A consequence of this overlap is that atypical forms of perception should influence working memory. We examined this by investigating whether having grapheme–color synesthesia, a condition characterized by the involuntary experience of color photisms when reading or representing graphemes, would confer benefits on working memory. Two competing hypotheses propose that superior memory in synesthesia results from information being coded in two information channels (*dual-coding*) or from superior dimension-specific visual processing (*enhanced processing*). We discriminated between these hypotheses in three *n*-back experiments in which controls and synesthetes viewed inducer and non-inducer graphemes and maintained color or grapheme information in working memory. Synesthetes displayed superior color working memory than controls for both grapheme types, whereas the two groups did not differ in grapheme working memory. Further analyses excluded the possibilities of enhanced working memory among synesthetes being due to greater color discrimination, stimulus color familiarity, or bidirectionality. These results reveal enhanced dimension-specific visual working memory in this population and supply further evidence for a close relationship between sensory processing and the maintenance of sensory information in working memory.

## Highlights

• Grapheme–color synesthetes display superior color working memory than controls.
• This effect is independent of color familiarity and color discrimination abilities.
• Controls and synesthetes do not differ in grapheme working memory.
• These results support enhanced color processing in synesthesia.
• They also support research linking sensory processing and working memory.

## 1 Introduction

Working memory allows the online maintenance of a limited amount of information in consciousness. There is emerging evidence that the encoding of sensory information and the maintenance of this information in a temporarily accessible state in working memory rely on the same neural mechanisms (Jonides, Lacey, & Nee, 2005; Postle, 2006; Serences, Ester, Vogel, & Awh, 2009). For instance, Serences et al. (2009) found that activation patterns in V1 during the maintenance of color and orientation information closely resembled those observed during the encoding of these features. A consequence of this overlap is that atypical forms of perception should influence working memory in the affected sensory modality. This idea can be explored in *grapheme–color synesthesia*, a form of idiosyncratic binding in which an individual involuntarily and reliably experiences color photisms (images or percepts; *concurrents*) when reading or representing numerals and letters (inducers; Grossenbacher & Lovelace, 2001; Rich & Mattingley, 2002; Ward, 2013).

Currently there is no clear evidence that synesthesia affects working memory. However, multiple case studies (Luria, 1968; Mills, Innis, Westendorf, Owsianiecki, & McDonald, 2006; Smilek, Dixon, Cudahy, & Merikle, 2002) and group studies (Gibson, Radvansky, Johnson, & McNerney, 2012; Gross, Neargarder, Caldwell-Harris, & Cronin-Golomb, 2011; Radvansky, Gibson, & McNerney, 2011; Rothen & Meier, 2010; Yaro & Ward, 2007) have documented superior episodic memory for inducer stimuli in synesthetes than in non-synesthete controls (for a review, see Rothen, Meier, & Ward, 2012). Insofar as working memory and long-term memory reciprocally facilitate each other (e.g., Baddeley, 2012), it is plausible that enhanced working memory among synesthetes may subserve superior episodic memory in this population.

Further evidence that synesthesia may affect working memory comes from studies showing that synesthesia impacts performance on selective attention tasks that include inducers (Dixon, Smilek, Cudahy, & Merikle, 2000; Dixon, Smilek, & Merikle, 2004; Mattingley, Rich, Yelland, & Bradshaw, 2001; Wollen & Ruggiero, 1983). For example, synesthetes are slower to identify the color of incongruently-colored than congruently-colored graphemes. Coupled with the recognition that the ability to selectively adjust attention is an important determinant of working memory capacity (Engle, 2002), synesthetes may experience interference costs on working memory from synesthetically-incongruent inducers, as has been found in word recall (Radvansky et al., 2011). However, a recent study found that synesthetes do not differ from controls in a standard Stroop color-naming task (Rouw, van Driel, Knip, & Ridderinkhof, 2013). Given the relationship between working memory and attentional control (Kane & Engle, 2003), this result suggests that synesthetes will not exhibit superior working memory, although it is possible that a synesthesia-specific working memory advantage is present for inducer or concurrent information. Although no model has specified predictions regarding working memory in synesthesia, such predictions can be derived from two competing hypotheses that have been advanced to explain the benefits of synesthesia to episodic memory.

According to the *dual coding hypothesis* (Paivio, 1969, 1986), superior memory may occur when associated verbal and color information is concurrently encoded because the coding of information in multiple slave systems may strengthen the representation of the information. In non-synesthetes, for instance, working memory capacity is greater when stimuli are presented bimodally (e.g., audio and visual) than unimodally (Mastroberardino, Santangelo, Botta, Marucci, & Olivetti Belardinelli, 2008). Thus, synesthetes may display superior memory because they have two associated channels by which a stimulus can be encoded and maintained. For example, a grapheme and its concurrent photism may be maintained separately in a phonological loop and a visual cache, respectively (e.g., Logie, 2011), allowing either representation to be subsequently used to classify stimuli as the same (targets) or different (foils). This hypothesis readily explains enhanced memory for inducers in synesthetes (Gross et al., 2011; Radvansky et al., 2011; Yaro & Ward, 2007), as well as self-reports that synesthetes explicitly use color photisms as a mnemonic aid (Pearce, 2007; Rich, Bradshaw, & Mattingley, 2005; Rothen & Meier, 2010; Yaro & Ward, 2007).

The dual-coding hypothesis makes clear predictions regarding the impact of congruency on working memory. Specifically, if the information in the two channels is congruent, working memory should be selectively enhanced in synesthetes, whereas if the information is incongruent, working memory maintenance should be weakened because of interference in the second channel. This prediction is consistent with the impact of synesthesia on selective attention, as described above. However, the benefits and hindrances conferred on *memory* by a dual-coding mechanism, as reflected in congruency effects on performance, are not always observed in synesthetes (e.g., Rothen & Meier, 2009; Yaro & Ward, 2007). Similarly, in contrast with the predictions of dual-coding theory, two studies found that synesthetes did not differ from controls (Gross et al., 2011) or a normative sample (Rothen & Meier, 2010) in digit span tasks, alternately interpreted as measures of short-term memory or working memory. However, insofar as both studies used verbal material, neither was able to examine congruency effects in working memory for inducer stimuli. An important corollary of this account is that if superior memory is facilitated by a second, ancillary sequence code, then any memory advantage will be restricted to the domain of the inducer and will not be observed with stimuli that do not elicit synesthetic color photisms. This prediction is at odds with the repeated observation that relative to non-synesthetes, synesthetes exhibit superior recognition memory for color stimuli that do not elicit synesthetic experiences (Rothen & Meier, 2010; Yaro & Ward, 2007).

The latter results suggest an alternative account, namely that enhanced modality- or dimension-specific processing among synesthetes facilitates superior memory in the *respective* modality or dimension. This *enhanced processing* hypothesis is supported by results showing that grapheme–color synesthetes exhibit superior low-level visual processing (Barnett et al., 2008), color discrimination (Banissy, Walsh, & Ward, 2009; Yaro & Ward, 2007), precision of color and luminance matching (Arnold, Wegener, Brown, & Mattingley, 2012), and color recognition memory (Yaro & Ward, 2007). Superior low-level visual processing may strengthen representations held in working memory by amplifying incoming sensory information or excluding external noise (Lu & Dosher, 2009) and thereby enhance maintenance of the information.^1^ For example, it has been shown that individual differences in color constancy, which enables stable color perception across different levels of illumination, is associated with individual differences in working memory (Allen, Beilock, & Shevell, 2011). Shared mechanisms underlying perception and memory (Chun & Johnson, 2011) similarly entail that enhanced color perception among synesthetes (Arnold et al., 2012; Banissy et al., 2009; Yaro & Ward, 2007) will translate to enhanced color working memory. Crucially, this account predicts enhanced working memory for color in synesthetes irrespective of whether the color functions as a concurrent. However, this hypothesis does not make explicit predictions regarding whether concurrent color photisms will benefit or hinder performance and thus it is agnostic regarding possible synesthetic congruency effects in working memory.

The goals of the present study were twofold. First, we examined whether synesthesia confers any benefits on working memory. Second, we sought to discriminate between the dual-coding and enhanced processing hypotheses as applied to working memory in synesthesia. The dual-coding hypothesis states that synesthetes will exhibit enhanced memory for inducer graphemes but not non-inducer graphemes. The enhanced processing hypothesis, on the other hand, holds that synesthetes will display superior low-level visual processing that may be specific to color. This account therefore predicts that synesthetes will display superior memory for colors, but not (necessarily) for graphemes.

To test these divergent predictions, grapheme–color synesthetes and non-synesthetes completed *n*-back tasks with colored inducer and non-inducer graphemes. In Experiment 1, participants responded whether or not the grapheme *color* was presented two or three trials back in the trial sequence whereas in Experiment 2, they responded whether or not the current *grapheme* was presented *n* trials back in the sequence. In both experiments we manipulated inducer grapheme–color pairs to investigate the presence of congruency effects on working memory, which are predicted by the dual-coding hypothesis, but not the enhanced processing hypothesis. To eliminate a confound of color familiarity in Experiment 1, we administered the non-inducer graphemes task with canonical colors in Experiment 3. We demonstrate that synesthetes display superior color, but not grapheme, working memory compared to non-synesthetes.

## 2 Experiment 1

In this experiment grapheme–color synesthetes and controls were instructed to hold grapheme *colors* in working memory. The enhanced processing hypothesis predicts superior color working for *both* inducer and non-inducer graphemes in synesthetes. In contrast, according to the dual-coding hypothesis, synesthetes and non-synesthetes should not differ in color working memory. However, color photisms experienced with inducer graphemes should facilitate superior performance for congruently-colored inducer graphemes and poorer performance for incongruently-colored inducer graphemes among synesthetes. The dual-coding hypothesis further predicts no advantage among synesthetes for non-inducer graphemes. To relate our results to previous findings, we also explored whether color working memory performance was related to individual differences in color discrimination ability (Banissy et al., 2009; Yaro & Ward, 2007).

Insofar as only preliminary research has been done on short-term memory and working memory in synesthesia (Gross et al., 2011; Meier & Rothen, 2010), it is unclear whether any observed group differences would manifest at information processing or decisional stages of task performance. For instance, performance differences across groups may reflect differences in the rate at which stimulus information is accumulated (*drift rate*), the amount of information required to make a response decision (*boundary separation*), or other nondecisional factors that influence responses (*nondecision time*) (Ratcliff, 1978; Wagenmakers, van der Maas, & Grasman, 2007). We investigated the processing locus of group differences by applying the EZ diffusion model (Wagenmakers et al., 2007), which incorporates different response components to estimate these three parameters for individual participants, as a supplement to conventional response accuracy and latency measures.

### 2.1 Method

#### 2.1.1 Participants

Sixteen controls (12 female, *M*_Age_ = 24.1, *SD* = 1.5) and 16 grapheme–color synesthetes (13 female, *M*_Age_ = 23.9, *SD* = 4.8) were recruited from the University of Oxford and consented to participate in accordance with approval from a local ethics committee. All participants were right-handed, had normal or corrected-to-normal vision, and were naïve to all hypotheses.

Information for digit–color consistency was available for 9 synesthetes. On two separate occasions separated by 32 days (range: 3–117, *SD* = 34), using the same monitor, synesthetes identified from a color palette the colors that most closely matched their color photisms for the digits 0 through 9 and the respective RGB values (0–255) were recorded. We computed consistency using the formula described by Eagleman, Kagan, Nelson, Sagaram, and Sarma (2007), for which lower values reflect greater consistency. All synesthetes displayed consistency values well below 1 (range: .11–.56; *M* = .24, *SD* = .14), which is considered diagnostic of genuine synesthesia (Eagleman et al., 2007); consistency was unrelated to the number of days between grapheme–color association tests, *r*_s_ = .10.

#### 2.1.2 Materials

##### 2.1.2.1 Working memory

All participants completed two *n*-back tasks with colored inducer graphemes and non-inducer graphemes. Trials consisted of colored graphemes presented centrally along the horizontal and vertical axes of a computer monitor against a gray background at a distance of approximately 70 cm, subtending a visual angle of 1.2–2.9° × 1.6–2.9°. Stimuli were drawn from eight different colored digits and eight different non-inducer colored graphemes comprised of familiar punctuation and mathematical symbols that did not elicit synesthetic colors (÷ = # ∞ ∗ ↔ ? %). The same colors were used for both stimulus types. Inducer graphemes were presented in a congruent (50% of trials) or an incongruent (50% of trials) color with respect to synesthetes’ color photisms. Within blocks, for each grapheme, each of the eight colors was presented twice as a target, twice as a predictor stimulus (a stimulus that predicts a target) and twice as a foil (a stimulus that does not correspond to the *n*-back trial). Stimulus presentation was randomized within blocks with the constraint that each block contained 33% targets and 67% foils. Control participants were randomly assigned to the stimulus set of a synesthete.

##### 2.1.2.2 Color discrimination

All participants completed the *Farnsworth–Munsell Color Hue Test* of color discrimination (Farnsworth, 1957). This task consists of four sequences of 23 or 24 color caps (blue, green, pink and yellow) that vary in hue but have identical luminance. For each set, the first and last caps were placed in the correct sequence positions and the intermediate caps were randomized; participants were given 2 min to arrange the caps. The score for each set was the sum of each hue’s deviation from the correct sequence and the mean set score formed the outcome measure (for further scoring information see Banissy et al., 2009).

#### 2.1.3 Procedure

**Fig. 1:**
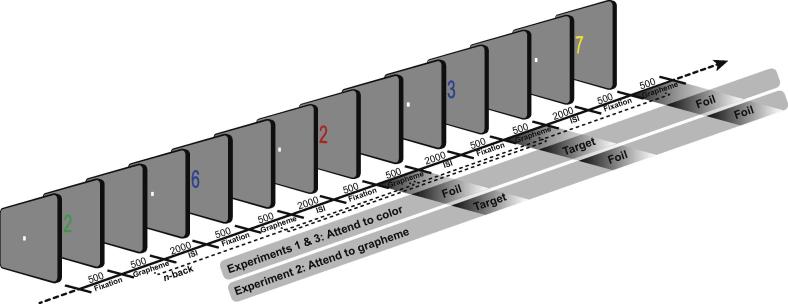
Schematic diagram of the task structure. Blocks consisted of a stream of stimuli in which participants attended to the color of a grapheme (Experiments 1 and 3) or the grapheme itself (Experiment 2) and responded whether the stimulus dimension was the same (Target) or different (Foil) as the stimulus dimension *n* trials back in the sequence. Stimuli were either inducer graphemes (depicted here) or non-inducer graphemes. Participants completed two-back (depicted here) and three-back tasks in separate blocks. Numbers reflect stimuli durations (ms). ISI = Interstimulus interval.

Prior to the experiment, synesthetes were interviewed to corroborate their synesthesia and determine their grapheme–color pairs. Synesthetes were required to experience unique colors for at least eight digits. The eight digits that elicited the strongest synesthetic experience (by self-report) comprised the stimulus set; symbols that did not elicit color photisms, by self-report, were selected as non-inducer graphemes. All non-synesthetes reported having no grapheme–color associations. Participants first completed 2-back and 3-back practice blocks (40 trials) for the inducer and non-inducer graphemes task and then eight experimental blocks (48 trials) of inducer graphemes and four blocks of non-inducer graphemes (alternating between 2-back and 3-back blocks (e.g., Kane, Conway, Miura, & Colflesh, 2007)). All trials began with a white fixation dot for 500 ms (see Fig. 1). The centrally-presented stimulus then appeared for 500 ms. This was followed by a blank 2000 ms interstimulus interval and then the next fixation dot. Participants were instructed to respond whether the current stimulus color matched the color presented either two or three back in the sequence by depressing one of two keys, corresponding to ‘yes’ and ‘no’ responses with the index and middle fingers of their right hand, using a Cedrus response pad (Cedrus Corporation, San Pedro, CA). The mapping of key to finger was counterbalanced across participants. Following completion of the tasks, participants completed the *Farnsworth–Munsell Color Hue Test*.

#### 2.1.4 Statistical analyses

We evaluated the dual-coding and enhanced processing hypotheses using error rates (ERs) and outlier-trimmed (±2 *SD*s) response times (RTs) in the two *n*-back tasks. In order to identify the processing locus of significant effects of relevance to the hypotheses under test, and disentangle different candidate mechanisms underlying group differences, we adopted a mathematical modeling approach using the EZ diffusion model (Wagenmakers et al., 2007). This model uses mean RT and RT variance on correct trials and ER to compute three parameters: *v* (drift rate), which indexes information accumulation and can be interpreted as a general measure of ability; *a* (boundary separation), which provides a measure of response conservativeness based on the volume of information required before a response will be provided; and *T*_er_ (nondecision time), which indexes nondecision (visual encoding and motor) processes. We performed a series of *EZ checks* (Wagenmakers et al., 2007) across conditions and working memory loads to examine whether the data met the assumptions of the EZ diffusion model: (1) positively-skewed RT distributions; (2) equivalent RTs on error and correct trials; and (3) no interaction between response (error vs. correct) and stimulus category (foil vs. target) on RTs. We tested these assumptions using Bonferroni-corrected ANOVAs (Wagenmakers et al., 2007) and we report the percentage of violations and any effects on the analyses.

Data were submitted to 2 (Load: 2-back vs. 3-back) × 2 (Type: foil vs. target) × 2 (Congruency: congruent vs. incongruent) × 2 (Group: controls vs. synesthetes) mixed-model analyses of variance (ANOVA). The Congruency factor was omitted in analyses of the non-inducer graphemes task. Subsidiary analyses of covariance (ANCOVAs) included Color discrimination scores as a covariate in order to investigate its influence on the observed effects. We report 95% confidence intervals for effect sizes $\left(\eta_{p}^{2}\right)$ for principal effects of direct relevance to the hypotheses under test.

Multiple Congruency × Group interactions were non-significant and thus we sought to clarify whether these effects more closely supported the dual-coding or the null hypotheses using a Bayesian approach. Specifically, we contrasted the magnitude of observed Congruency effects in synesthetes against that predicted by the dual-coding hypothesis using the Bayes factor (*B*; Dienes, 2011), which indexes the likelihood of a hypothesis, relative to the null, given a set of data and thereby permits a more robust comparison between a tested hypothesis and the null than orthodox statistics. *B* values greater than 1 indicate that the data support the tested hypothesis over the null, whereas values below 1 indicate support for the null. Jeffreys (1961) further proposes that values between 0 and .33 should be interpreted as reflecting strong support for the null over the tested hypothesis; values between .33 and 3 should be regarded as inconclusive; and values greater than 3 should be interpreted as clearly supporting the hypothesis under test (see also Dienes, 2011). For these computations we used the *M* and *SEM* of the magnitude of the Congruency effect (incongruent–congruent) in ERs in the synesthetes and contrasted these with values predicted by the dual-coding hypothesis. We expected that synesthesia-specific Congruency effects in working memory driven by dual-coding processes should be comparable to those observed in a recall task that included inducer words that were either congruent or incongruent relative to grapheme–color synesthetes’ photism colors (Radvansky et al., 2011). The sample size (*n* = 10) and gender distribution (eight females) of the synesthetes in the latter study were comparable to those of our samples in Experiments 1 and 2 (age data were not available for the Radvansky et al. (2011) sample, although all participants were university students). We computed the magnitude of the Congruency effect (incongruent ER–congruent ER) in Radvansky et al.’s (2011) study (*M*_SynCong_ = .08, *SD* = .05) and included this as the predicted size of the Congruency effect in our computations of *B* (two-tailed). Along with *B*, we report the mean (*M*_SynCong_) and standard error (*SEM*) of the respective Congruency effect.

### 2.2 Results

#### 2.2.1 Color discrimination

Synesthetes (*M* = 36.63, *SD* = 14.27) exhibited numerically, albeit non-significantly, greater color discrimination than the controls (*M* = 45.50, *SD* = 21.93), unequal variance *t*(25.77) = 1.36, *p* > .05 (lower scores reflect superior performance), although the effect size, *d* = .50, is not markedly lower than those in previous studies (*d* = .65 [Banissy et al., 2009]; *d* = .69 [Yaro & Ward, 2007]).^2^

#### 2.2.2 *n*-Back tasks

Descriptive statistics for performance on the *n*-back tasks in all experiments are presented in Table 1.

##### 2.2.2.1 Error rates

In the inducer graphemes *n*-back, there was a main effect of Load, *F*(1, 30) = 75.90, *MSE* = 0.02, *p* < .001, $\eta_{p}^{2}=.72$, and a suggestive main effect of Type, *F*(1, 30) = 3.99, *MSE* = 0.34, *p* = .055, $\eta_{p}^{2}=.12$, which were qualified by a Load × Type interaction, *F*(1, 30) = 12.68, *MSE* = 0.01, *p* = .001, $\eta_{p}^{2}=.30$, reflecting greater ERs for targets than foils in the 3-back condition, *F*(1, 31) = 8.51, *MSE* = 0.04, *p* = .007, $\eta_{p}^{2}=.22$, but not in the 2-back condition, *F* < 0.5. In addition, a main effect of Congruency, *F*(1, 30) = 4.74, *MSE* < 0.01, *p* = .038, $\eta_{p}^{2}=.14$, revealed that participants performed better in the congruent than in the incongruent condition. In contrast with the dual-coding hypothesis, there was no Congruency × Group interaction, *F* < 0.01, $\eta_{p}^{2}<.01$ (95% CIs: .00, .01). To clarify the main effect of Congruency, we performed exploratory ANOVAs separately in each group. This effect was not independently present in controls, *F* < 2.6, or synesthetes, *F* < 2.3. Crucially, the magnitude of the Congruency effect in synesthetes was inconsistent with the prediction of the dual-coding hypothesis, *M*_SynCong_ = .017, *SEM* = .011, *B* = .33.

**Fig. 2:**
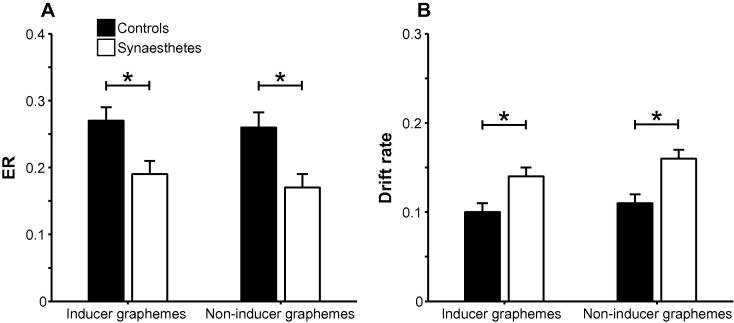
Means ±1 standard error of the mean (SEM) for (A) ER and (B) drift rate in the inducer and non-inducer graphemes tasks in controls and synesthetes in Experiment 1 ^*^p < .05.

As predicted by the enhanced processing hypothesis, there was a main effect of Group**,** *F*(1, 30) = 6.78, *MSE* = 0.06, *p* = .014, $\eta_{p}^{2}=.18$ (95% CIs: .01, .40), with synesthetes displaying lower ERs than controls (see Fig. 2A). An inspection of the descriptive statistics in Table 1 suggests that this effect may be driven by especially high error rates for 3-back targets in controls, but importantly there were no Group interactions involving Load or Type, *F*s < 1, and no other main effects or interactions, *F*s < 3.8. The main effect of Group was replicated when Color discrimination was included as a covariate, *F*(1, 29) = 4.73, *MSE* = 0.05, *p* = .038, $\eta_{p}^{2}=.14$, with Color discrimination exerting a suggestive independent effect, *F*(1, 29) = 3.76, *MSE* = 0.05, *p* = .062, $\eta_{p}^{2}=.12$, reflecting a positive correlation between color discrimination values and ERs in the *n*-back task, *r* = .40, *p* = .023, *r*_p_ = .34 (controlling for group).

In the non-inducer graphemes task there was a main effect of Load, *F*(1, 30) = 42.01, *MSE* = 0.01, *p* < .001, $\eta_{p}^{2}=.58$, and a Load × Type interaction, *F*(1,30) = 17.72, *MSE* = 0.01, *p* < .001, $\eta_{p}^{2}=.37$. As with the inducer graphemes, this reflected greater ERs for targets than foils in the 3-back condition, *F*(1, 30) = 8.63, *MSE* = 0.02, *p* = .006, $\eta_{p}^{2}=.23$, but not in the 2-back condition, *F* < 3.1. Crucially, as with the inducer graphemes task, we also found a main effect of Group, *F*(1, 30) = 9.82, *MSE* = 0.03, *p* = .004, $\eta_{p}^{2}=.25$ (95% CIs: .03, .46), with synesthetes performing better than controls (see Fig. 2). There were no other effects, *F*s < 2.3. The Group effect was stable when Color discrimination was treated as a covariate, *F*(1, 29) = 7.62, *MSE* = 0.03, *p* = .010, $\eta_{p}^{2}=.21$, but Color discrimination did not exhibit an independent effect, *F* < 1.8, *r*_p_ = .24. However, this correlation was suggestive when Group was not included as a covariate, *r* = .32, *p* = .072.

##### 2.2.2.2 Response times

**Fig. 3:**
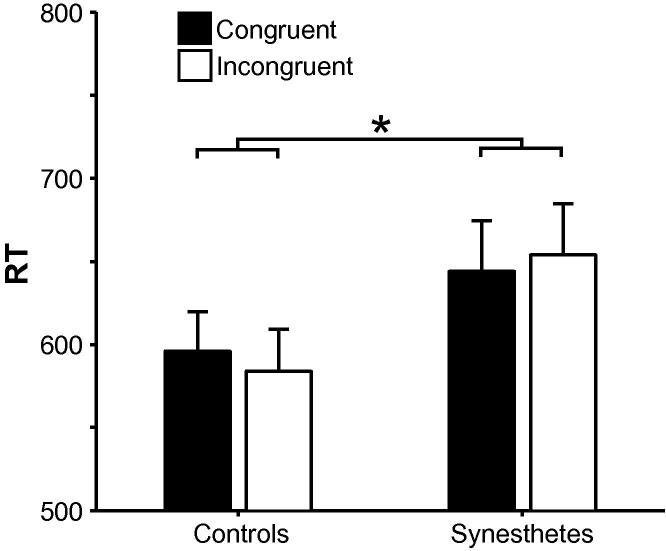
Mean RT ±1 standard error of the mean (SEM) in the inducer graphemes task as a function of Congruency in controls and synesthetes in Experiment 1 ^*^p < .05.

In the inducer graphemes task, main effects of Load, *F*(1, 30) = 4.28, *MSE* = 18,809, *p* = .047, $\eta_{p}^{2}=.13$, and Type, *F*(1, 30) = 12.09, *MSE* = 8,508, *p* = .002, $\eta_{p}^{2}=.29$, were qualified by Load × Type, *F*(1, 30) = 4.54, *MSE* = 1,980, *p* = .041, $\eta_{p}^{2}=.13$, and Load × Congruency × Type interactions, *F*(1, 30) = 6.26, *MSE* = 1,380, *p* = .018, $\eta_{p}^{2}=.17$. The Congruency × Type interaction was significant in the 2-back condition, *F*(1, 30) = 5.60, *MSE* = 843, *p* = .025, $\eta_{p}^{2}=.16$ (the Congruency effect was not significant for foils or targets, *F*s < 3.5), but not in the 3-back condition, *F* < 1.7. There was also a Congruency × Group interaction, *F*(1, 30) = 4.24, *MSE* = 1,882, *p* = .048, $\eta_{p}^{2}=.12$ (95% CIs: .00, .34): the Congruency effect was greater among synesthetes than controls (see Fig. 3), but neither effect was independently significant, synesthetes: *F* < 2.4, *η*^2^ = .14 (95% CIs: .00, .42), controls: *F* < 2.^3^ No other effects were found, *F*s < 3.2. Color discrimination, when included as a covariate, did not exhibit an effect on task performance, *F* < 0.04.

In the non-inducer graphemes task, the main effects of Load *F*(1, 30) = 5.40, *MSE* = 9,041, *p* = .027, $\eta_{p}^{2}=.15$, reflecting faster RTs in the 2-back than in the 3-back condition, and Type, *F*(1, 30) = 10.07, *MSE* = 5623, *p* = .003, $\eta_{p}^{2}=.25$, reflecting faster RTs for targets than foils, were replicated, but there were no other effects, *F*s < 3.2. Again, when color discrimination was included as a covariate, it did not affect task performance, *F* < 0.02.

##### 2.2.2.3 Diffusion modeling

Synesthetes displayed significantly lower ERs than controls, but (non-significantly) slower RTs, suggesting the former effect may reflect, at least partially, a speed-accuracy tradeoff. To address this possibility, and to investigate the information processing locus of group effects, we applied the EZ diffusion model (Wagenmakers et al., 2007) to accuracy and RT data. Analyses of the diffusion modeling parameters revealed that synesthetes exhibited greater drift rates, reflecting superior information accumulation, in both the inducer graphemes task, *F*(1, 30) = 7.95, *MSE* = 0.02, *p* = .008, $\eta_{p}^{2}=.21$ (95% CIs: .02, .43), and the non-inducer graphemes task, *F*(1, 30) = 10.07, *MSE* = 0.01, *p* = .003, $\eta_{p}^{2}=.25$ (95% CIs: .03, .46) (see Fig. 2B). There were no other Group effects for any of the diffusion parameters in either task, *F*s < 3.8, indicating that greater information accumulation among synesthetes was not enabled by differential decision boundaries or nondecisional slowing of responses. Crucially, there were no Congruency × Group interactions in drift rate, *F* < 2.9, $\eta_{p}^{2}=.09$ (95% CIs: .00, .30), boundary separation, *F* < 2.6, $\eta_{p}^{2}=.08$ (95% CIs: .00, .29), or nondecision time, *F* < 0.08, $\eta_{p}^{2}=.00$ (95% .00, .09). Similarly, including color discrimination as a covariate did not alter the results and it did not exhibit an independent significant effect on any of the parameters, *F*s < 2.2. EZ checks of the assumptions of the EZ diffusion model revealed that the three model assumptions were violated by 1%, 2%, and 4% of the data, respectively. The results were unaffected when participants with data that violated one or more assumptions were excluded from the analyses.

### 2.3 Discussion

Grapheme–color synesthetes exhibited superior color working memory than non-synesthetes. This effect was comparable in size for inducer ($\eta_{p}^{2}$ range: .18–.21) and non-inducer ($\eta_{p}^{2}$ range: .25) graphemes and thus was not specific to synesthetic inducers. Both effects were restricted to drift rate, thereby suggesting that synesthetes’ enhanced performance reflects superior uptake of information and greater stimulus classification when making a response. The difference between synesthetes and controls appears to occur at the stimulus processing stage, which is very much consistent with the enhanced processing hypothesis. Moreover, these results also indicate that synesthetes’ superior performance is independent of a possible speed-accuracy tradeoff because the two groups did not differ in response latencies or any other diffusion parameters. The results provide clear support for the enhanced processing hypothesis and are consistent with previous results showing superior color processing in this population (Arnold et al., 2012; Banissy et al., 2009; Yaro & Ward, 2007).

We further examined whether enhanced color working memory among synesthetes is driven by superior color discrimination. Although we failed to replicate the finding of superior color discrimination in this group (Banissy et al., 2009; Yaro & Ward, 2007), the observed difference was in the predicted direction and the effect size was not markedly lower than that found in previous studies. Indeed, the synesthetes actually performed at a comparable level to those in previous studies, whereas our controls outperformed the controls in previous studies. Importantly, the advantage of synesthesia in color working memory was independent of individual differences in color discrimination, which only suggestively affected performance in the two tasks.

The dual-coding hypothesis predicts superior performance for inducer graphemes among synesthetes, particularly inducers that are congruently-colored. We observed two results relating to this prediction. First, in ERs, there was a main effect of Congruency on ERs across both Groups. This somewhat unexpected finding may have resulted from the large proportion of congruent trials in the inducer grapheme task. We included equivalent proportions of congruent and incongruent trials, which is known to increase the magnitude of the Stroop effect (Macleod, 1991), in order to augment our ability to detect a synesthetic Congruency effect. However, given the proportion of congruent trials, each of the individual congruent grapheme–color pairs were presented more frequently than each of the individual incongruent grapheme–color pairs, participants may have implicitly learned to associate graphemes and colors that were more frequently paired than those that were paired less frequently in what amounts to a contingency learning artifact (see, e.g., Schmidt & Besner, 2008). However, our assessment of whether the synesthetic Congruency effect supports the prediction of the dual-coding hypothesis using the Bayes factor (Dienes, 2011) was restricted to synesthetes and thus is not hindered by this confound. The corresponding Bayes factor was .33 and is consistent with the null hypothesis that there was no Congruency effect in response accuracy in synesthetes. Synesthetes did display a greater congruency effect in response latencies than controls. Crucially, this effect reflected suggestively slower RTs for incongruent trials among synesthetes, but no advantage for congruent trials. This result is clearly at odds with the dual-coding hypothesis (Paivio, 1969, 1986), which predicts a specific advantage for congruent trials. Rather, it seems that color photisms on incongruent trials elicit greater response conflict and thereby delay responses whereas photisms on congruent trials neither advantage nor disadvantage working memory performance.^4^ Cumulatively, these results are inconsistent with the dual-coding hypothesis.

## 3 Experiment 2

Experiment 1 provided support for the enhanced processing hypothesis but did not constitute a stringent test of the dual-coding hypothesis because participants maintained colors, not graphemes, in working memory. To more clearly test the prediction that synesthetic colors facilitate maintenance of graphemes in working memory, participants completed the same *n*-back tasks, but responded as to whether the current *grapheme* had previously been presented in the sequence. If a dual-coding mechanism contributes to working memory maintenance in synesthesia, then synesthetes should exhibit superior working memory for inducer graphemes, particularly those that are congruently-colored, than non-synesthetes, but not for non-inducer graphemes.

### 3.1 Method

#### 3.1.1 Participants

Ten controls (9 female, *M*_Age_ = 24.4, *SD* = 1.58) and 10 grapheme–color synesthetes (9 female, *M*_Age_ = 22.4, *SD* = 4.2), recruited from the University of Oxford, participated. Nine synesthetes and 8 controls took part in Experiment 1; the two experiments were conducted 2 months apart. The same synesthetes were included in this study because it is challenging to recruit new synesthetes; the same controls were included because we wanted the groups to be relatively matched on any performance advantages conferred by participating in multiple working memory experiments.

Digit–color consistency information was available for 4 synesthetes. The procedure and analysis was the same as that reported in Experiment 1. Digit–color associations were recorded on two separate days separated by 42 days (range: 3–117, *SD* = 53) and all synesthetes displayed consistency values (range: .11–.56; *M* = .24, *SD* = .14) considered to be diagnostic of synesthesia (Eagleman et al., 2007); consistency was unrelated to the number of days between grapheme–color association tests, *r*_s_ = .00.

#### 3.1.2 Design and procedure

Participants completed the same inducer and non-inducer graphemes *n*-back tasks as in Experiment 1 with two changes: (1) participants were instructed to respond whether the current *grapheme* matched that which was presented two or three steps back in the sequence; and (2) the non-inducer graphemes task used achromatic stimuli.

### 3.2 Results

#### 3.2.1 Error rates

**Fig. 4:**
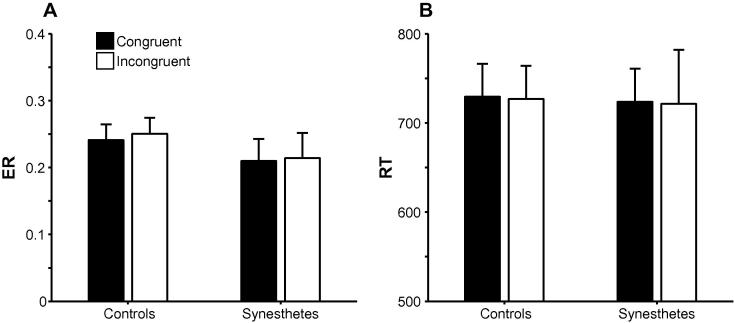
Means ±1 standard error of the mean (SEM) for (A) ER and (B) RT in the inducer graphemes task as a function of Congruency in controls and synesthetes in Experiment 2.

There was a main effect of Load in the inducer graphemes task, *F*(1, 18) = 24.12, *MSE* = 0.02, *p* < .001, $\eta_{p}^{2}=.57$, with participants making fewer errors in the 2-back than in the 3-back condition. Crucially, there were no main effects of Congruency, *F* < 0.5, or Group, *F* < 0.7, or a Congruency × Group interaction, *F* < 0.07, $\eta_{p}^{2}=.00$ (95% CIs: .00, .12) (see Fig. 4A). As in Experiment 1, the synesthetes’ Congruency effect, *M*_SynCong_ = –.004, *SEM* = .012, was inconsistent with the prediction of the dual-coding hypothesis, *B* = .07; there were no other effects, *F*s < 2.7.

In the non-inducer graphemes task, main effects of Load, *F*(1, 18) = 38.91, *MSE* = 0.01, *p* < .001, $\eta_{p}^{2}=.68$, and Type, *F*(1, 18) = 7.92, *MSE* = 0.02, *p* = .011, $\eta_{p}^{2}=.31$, were qualified by a Load × Type interaction, *F*(1, 18) = 12.12, *MSE* = 0.01, *p* = .003, $\eta_{p}^{2}=.40$. This was driven by lower ERs for foils than targets in the 3-back condition, *F*(1, 19) = 12.72, *MSE* = 0.02, *p* = .002, $\eta_{p}^{2}=.40$, but not in the 2-back condition, *F* < 1. There was no main effect of Group, *F* < 1.3, or any other effects, *F*s < 1.3.

The dual-coding hypothesis predicts that synesthetes should display superior maintenance for congruently-colored graphemes than non-inducer graphemes. We examined this by including congruent inducer graphemes and non-inducer graphemes as the two levels of a Congruency factor in an ANOVA that also included Load, Type, and Group as independent variables. This analysis again failed to find a Congruency × Group interaction, *F* < 0.4, $\eta_{p}^{2}=.02$ (95% CIs: .00, .24).

#### 3.2.2 Response times

In the inducer graphemes task, there were main effects of Load, *F*(1, 18) = 4.99, *MSE* = 17,914, *p* = .038, $\eta_{p}^{2}=.22$, reflecting slower RTs in the 3-back condition, and Type, *F*(1, 18) = 9.07, *MSE* = 8,531, *p* = .007, $\eta_{p}^{2}=.34$, reflecting slower RTs for foils. Crucially, in contrast with dual coding theory, there were neither main effects of Congruency, *F* < 0.5, or Group, *F* < 0.1, nor a Congruency × Group interaction, *F* < 0.1, $\eta_{p}^{2}=.00$ (95% CIs: .00, .00) (see Fig. 4B). There was a Load × Type × Group interaction, *F*(1, 18) = 4.67, *MSE* = 3,310, *p* = .044, $\eta_{p}^{2}=.21$; controls exhibited a numerically, but non-significantly, greater Load effect than synesthetes on foils, Load × Group: *F* < 2.4, but not on targets, *F* < 0.01. There was also a Load × Congruency × Type interaction, *F*(1, 18) = 5.71, *MSE* = 1,685, *p* = .028, $\eta_{p}^{2}=.24$, reflecting different Congruency × Type interactions in the different load conditions; again, neither was independently significant, *F*s < 3.4. There were no other effects, *F*s < 2.9.

In the non-inducer graphemes task, there was a main effect of Load, *F*(1, 18) = 6.65, *MSE* = 10,123, *p* = .019, $\eta_{p}^{2}=.27$, reflecting slower RTs in the 3-back condition, and a suggestive effect of Type, *F*(1, 18) = 3.54, *MSE* = 6,106, *p* = .076, $\eta_{p}^{2}=.16$. There was no effect of Group, *F* < 0.1, or any other effects, *F*s < 3. When RTs for congruent and non-inducer graphemes were contrasted in an ANOVA, there was again no Congruency × Group interaction, *F* < 0.1, $\eta_{p}^{2}=.01$ (95% CIs: .00, .15).

#### 3.2.3 Diffusion modeling

It is possible that response accuracy and latency measures are not sufficiently sensitive to detect Group effects or Congruency × Group interactions in the inducer grapheme *n*-back task. To investigate this possibility, we applied diffusion modeling to the data as in Experiment 1. Again, there were no main effects of Group on drift rate, *F* < 0.8, boundary separation, *F* < 0.4, or nondecision time, *F* < 0.1. Crucially, there were also no Congruency × Group interactions on drift rate, *F* < 0.8, $\eta_{p}^{2}=.04$ (95% CIs: .00, .29), boundary separation, *F* < 0.1, $\eta_{p}^{2}=.00$ (95% CIs: .00, .09), or nondecision time, *F* < 1.8, $\eta_{p}^{2}=.09$ (95% CIs: .00, .36). In the non-inducer graphemes task, there were no main effects of Group on any of the diffusion parameters, *F*s < 1.9. However, there was a suggestive Load × Group interaction on nondecision time, *F*(1, 18) = 3.51, *p* = .077, $\eta_{p}^{2}=.16$ (95% CIs: .00, .43), reflecting a greater Load effect among controls, *F*(1, 9) = 15.81, *p* = .003, $\eta_{p}^{2}=.64$ (95% CIs: .12, .80) than synesthetes, *F* < 0.1.

Interestingly, there was a Load × Group interaction on boundary separation in the non-inducer graphemes task, *F*(1, 18) = 16.23, *MSE* < 0.01, *p* < .001, $\eta_{p}^{2}=.47$ (95% CIs: .12, .67), which was qualified by a Load × Type × Group interaction, *F*(1, 18) = 4.61, *MSE* < 0.01, *p* = .046, $\eta_{p}^{2}=.20$ (95% CIs: .00, .47). Controls displayed numerically higher boundary separation for foils than targets in the two-back condition, but lower values for foils than targets in the three-back condition, whereas synesthetes displayed the converse pattern. The Load × Type interaction did not achieve significance in either group, *F*s < 2.9. As in the inducer graphemes task, there was a Load × Group interaction on nondecision time, *F*(1, 18) = 8.23, *p* = .010, $\eta_{p}^{2}=.31$ (95% CIs: .02, .56), again reflecting a greater Load effect among controls, *F*(1, 9) = 38.85, *p* < .001, $\eta_{p}^{2}=.81$ (95% CIs: .38, .89), than synesthetes, *F* < 0.4. When diffusion parameters for congruent inducer and non-inducer graphemes were compared, there were no Congruency × Group interactions on drift rate: *F* < 0.4, $\eta_{p}^{2}=.02$ (95% CIs: .00, .24), boundary separation: *F* < 0.1, $\eta_{p}^{2}=.01$ (95% CIs: .00, .16), or nondecision time: *F* < 0.8, $\eta_{p}^{2}=.04$ (95% CIs: .00, .29). There were no other Group effects for any of the diffusion parameters in either task, *F*s < 3.6. As in Experiment 1, violations of the three assumptions of the EZ diffusion model were infrequent: 8%, 7%, and 3%, respectively. When the participants with data that violated these assumptions were excluded from the analyses, the Load × Group interaction on nondecision time was non-significant, *F* < 0.4, as was the Load × Type × Group interaction on boundary separation, *F* < 0.3, whereas the other interactions reported above remained significant.

### 3.3 Discussion

Grapheme–color synesthetes and non-synesthetes did not systematically differ in grapheme working memory. The central prediction of the dual-coding account is that synesthetes should display a greater stimulus-photism color Congruency effect, reflecting superior performance on congruent trials, than controls. This Congruency × Group interaction was not found in ERs, RTs, or three diffusion parameters.^5^ In a series of exploratory analyses contrasting these dependent variables in the congruent and non-inducer conditions, we similarly found no Congruency × Group interactions. The fact that the mean effect size for this interaction across the different analyses was .02 strongly indicates that our inability to detect this effect is not due to insufficient statistical power. This finding is crucial because effect size is independent of sample size (e.g., Fritz, Morris, & Richler, 2012) and thus even with a substantially larger sample size, this effect size would still not yield a statistically significant result. Consistent with this, the computed Bayes factors for the synesthetic Congruency effect in ERs (.07) provides substantial evidence for the null hypothesis. Further evidence that this study was not underpowered is marshaled by two other studies that did not observe superior digit span performance for inducer stimuli in synesthetes despite having larger sample sizes than the present study (*N* = 26 [controls: *n* = 20; synesthetes: *n* = 6]; Gross et al., 2011; *N* = 44 [synesthetes]; Rothen & Meier, 2010). It could be argued that synesthetic phosphenes on incongruent trials may afford cues that also aid memory and thus we should not expect congruency effects. However, such an interpretation still predicts a dual-coding driven overall advantage in inducer grapheme working memory among synesthetes, which we did not observe.

The only observed Group difference was a larger effect of working memory load on nondecision time in controls than synesthetes. This suggests that the higher working memory load did not tax encoding or motor preparatory processes as much in synesthetes and points to a suggestive advantage in this group that is specific to nondecisional processes. Crucially, this effect was only reliably observed for non-inducer graphemes and thus does not point to any specific advantage conferred by the online experience of synesthesia on working memory among synesthetes.

The failure to find support for the dual-coding hypothesis is consistent with our inability to detect an advantage among synesthetes for congruent graphemes in Experiment 1. Synesthetic congruency effects have been inconsistently observed in episodic memory tasks (Gibson et al., 2012; Radvansky et al., 2011; Rothen & Meier, 2009; Yaro & Ward, 2007) even though they have been repeatedly observed in selective attention tasks with inducer stimuli (Dixon et al., 2000, 2004; Wollen & Ruggiero, 1983). Cumulatively, these results indicate that experiencing ancillary color photisms during the encoding of graphemes does not confer any benefit in maintaining and updating grapheme sequences in working memory or manipulating such information in immediate memory (Gross et al., 2011; Rothen & Meier, 2010). As with those of Experiment 1, these results are therefore inconsistent with the dual-coding hypothesis as applied to working memory in synesthesia.

## 4 Experiment 3

One potential confound in Experiment 1 is differential color familiarity across groups. The stimulus colors were the same colors that synesthetes experience on a regular basis whereas these colors may have been relatively novel for non-synesthetes (e.g., mauve) and thus difficult to verbally code or maintain in working memory. In turn, differential stimulus color familiarity may have conferred a performance advantage for synesthetes and produced the observed group differences. A further potential limitation of Experiments 1 and 2 is that synesthesia was confirmed by self-report in an interview and not with behavioral measures of automaticity or consistency of grapheme–color associations, which are widely regarded as markers of genuine synesthesia (Ward, 2013). This experiment circumvents these limitations by replicating Experiment 1 using canonical colors that would be equally familiar to both groups and by verifying the consistency of synesthetes’ grapheme–color associations.

### 4.1 Method

#### 4.1.1 Participants

Eight controls (six female, *M*_Age_ = 25.38, *SD* = 4.21) and eight grapheme–color synesthetes (six female, *M*_Age_ = 25.13, *SD* = 3.80), recruited from the University of Oxford, participated. None had participated in Experiments 1 or 2.

Digit–color consistency information was available for all 8 synesthetes. The procedure and analysis was the same as that reported in Experiments 1 and 2. Digit–color associations were recorded on two separate days separated by 40 days (range: 9–100, *SD* = 32) and all synesthetes displayed consistency values (range: .17–.30; *M* = .22, *SD* = .04) considered to be diagnostic of synesthesia (Eagleman et al., 2007); consistency was unrelated to the number of days between grapheme–color association tests, *r*_s_ = −.002.

#### 4.1.2 Design and procedure

The non-inducer graphemes task of Experiment 1 was used in this experiment but grapheme colors were comprised of eight canonical colors (blue, orange, red, yellow, pink, purple, brown, and green) instead of synesthetes’ photism colors. Participants completed two practice and six experimental blocks (three 2-back and three 3-back). The experiment was conducted by a different experimenter than in Experiments 1 and 2.

### 4.2 Results

The data of one synesthete, who was a RT outlier in three of the four conditions (*Z*s > 1.96), were excluded from the analyses.

#### 4.2.1 Error rates

A mixed-model ANOVA revealed a main effect of Load, *F*(1, 13) = 47.34, *MSE* = 0.01, *p* < .001, $\eta_{p}^{2}=.79$, reflecting lower error rates in the 2-back than in the 3-back condition. There was no main effect of Group, *F* < 1.4, $\eta_{p}^{2}=.10$ (95% CIs: .00, .40), or any other effects, *F*s < 1.6.

#### 4.2.2 Response times

**Fig. 5:**
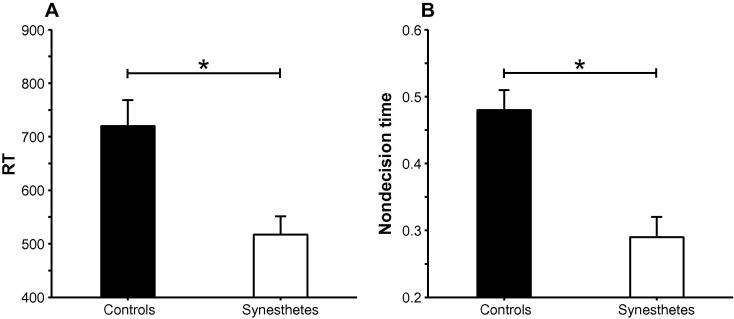
Means ±1 standard error of the mean (SEM) for (A) RT and (B) nondecision time in the non-inducer graphemes task in controls and synesthetes in Experiment 3 ^*^p < .01.

We again found a main effect of Load, *F*(1, 13) = 11.35, *MSE* = 5,267, *p* = .005, $\eta_{p}^{2}=.47$, with faster RTs in the 2-back than in the 3-back condition, but also a main effect of Group, *F*(1, 13) = 10.94, *MSE* = 56,194, *p* = .006, $\eta_{p}^{2}=.46$ (95% CIs: .05, .68), reflecting faster RTs among synesthetes than controls (see Fig. 5A). There were no other effects, *F*s < 3.7.

#### 4.2.3 Diffusion modeling

The analyses revealed that the Group effect was present in nondecision time (*T*_er_), *F*(1, 13) = 17.46, *MSE* = 0.03, *p* = .001, $\eta_{p}^{2}=.57$ (95% CIs: .13, .75), with synesthetes displaying lower values than controls (see Fig. 5B). There was no effect of Group on drift rate, *F* < 1.7, $\eta_{p}^{2}=.11$ (95% CIs: .05, .42), or boundary separation, *F* < 0.1, $\eta_{p}^{2}=.00$ (95% CIs: .00, .07), and no other Group effects, *F*s < 4.6. Violations of the three EZ diffusion model assumptions occurred in 0%, 7%, and 3% of the data, respectively, and the results were not affected when participants with data that violated these assumptions were excluded from the analyses.

### 4.3 Discussion

As in Experiment 1, synesthetes displayed superior color working memory than controls, even though the stimulus set was comprised of canonical colors. In Experiment 1, this effect was found in drift rate, suggesting that synesthetes displayed superior information accumulation and stimulus classification, whereas in this experiment it was present in nondecision time, suggesting that synesthetes displayed more efficient visual encoding (Wagenmakers et al., 2007). This discrepancy in the locus of group differences across experiments may be due to differential implicit emphasis of latency (Experiment 1) or accuracy (Experiment 3) by the different experimenters (Pachella, 1974) and concomitant differences in stimulus classification or visual encoding, respectively. Nevertheless, these results extend those of Experiment 1 and indicate that superior color working memory among synesthetes is neither an artifact of increased color familiarity nor reflective of enhanced working memory for colors that is *specific* to concurrent colors. Moreover, this Experiment reveals that superior color working memory can be replicated in an independent sample of participants whose synesthesia has been confirmed by behavioral testing.

An alternative explanation for enhanced color working memory among synesthetes is that color stimuli implicitly triggered numerical representations, which, in turn, aided maintenance of color sequences in working memory, as would be predicted by dual-coding theory (see, e.g., Rothen et al., 2012). Multiple studies have documented implicit, and even explicitly, bidirectionality in synesthetes (Cohen Kadosh, Cohen Kadosh, & Henik, 2007; Cohen Kadosh & Henik, 2006; Cohen Kadosh, Tzelgov, & Henik, 2008; Cohen Kadosh et al., 2005; Gebuis, Nijboer, & Van der Smagt, 2009; Gevers, Imbo, Cohen Kadosh, Fias, & Hartsuiker, 2010; Johnson, Jepma, & de Jong, 2007; Knoch, Gianotti, Mohr, & Brugger, 2005) and so this explanation is plausible. However, a number of results in this study are at odds with this interpretation. If bidirectionality is driving enhanced color working memory among synesthetes, performance should be superior for stimulus colors (e.g., navy blue) that are close in color space to a color photism for a particular numeral (e.g., light blue) than for stimulus colors that are greater in distance from the nearest photism color. We tested this prediction by computing the Euclidean distance between the stimulus color on each trial and the *nearest* photism color (based on the respective participant’s digit–color associations) and included color distance as a predictor of performance. For each participant, we performed multivariate linear regression on RTs and binary multivariate logistic regression on accuracy (incorrect = 0; correct = 1) with Load (2- vs. 3- back block), Trial type (foil vs. target), and Stimulus-photism color distance as predictors (forced entry regression method). The sample size of each regression analysis was 273, corresponding to the number of trials (excluding practice and the 1st 2 trials in 2-back blocks and the 1st 3 trials in 3-back blocks for which participants cannot make 2-back or 3-back judgments) (for a similar approach, see Notebaert & Verguts, 2007). In line with our results, Load significantly predicted RTs in 3 out of 7 participants (betas [*M* ± *SE*]: 79 ± 30) and accuracy in 5 participants, −0.80 ± 0.35. Trial type did not predict RTs in a single participant, −30 ± 10, but did predict accuracy in 2 participants, −0.47 ± 0.24. Crucially, Color distance predicted both RTs and accuracy in only a single synesthete, RT: 0.10 ± 0.14; accuracy: 0.01 ± 0.01. These results corroborate the effects of Load on performance (e.g., higher load being associated with slower RTs and more errors), and Trial type to a lesser extent, but further indicate that the distance between the stimulus color and the nearest photism color does not reliably predict performance. These results, in turn, strongly suggest that superior color working memory in synesthesia is not driven by bidirectionality.

Two further results are inconsistent with a bidirectionality account. If superior color working memory in synesthesia were driven by bidirectionality, we should expect larger effects in Experiment 1 than 3, because the former included synesthetic photism colors, which should implicitly trigger grapheme representations more than canonical colors. We did not observe this result. Furthermore, number–color associations are typically more robust in synesthetes than color–number associations; at the very least, the size of their impact on behavior is roughly equivalent (Gebuis et al., 2009). Thus, if superior color working memory were driven by bidirectionality, we would still expect synesthetes to display superior grapheme working memory (at least for congruent trials) than controls. Again, we did not observe this result in Experiment 2. These results strongly indicate that bidirectionality is not driving superior color working memory among synesthetes.

## 5 General discussion

This study examined whether grapheme–color synesthesia confers an advantage on visual working memory and sought to discriminate between two possible explanations for this effect. Synesthetes displayed superior color, but not grapheme, working memory than non-synesthetes. Crucially, this effect was present (and comparable in size) irrespective of whether the colored grapheme elicited synesthesia, thus demonstrating that it is not specific to the online experience of synesthesia. Furthermore, we show that enhanced color working memory among synesthetes is neither an artifact of superior color discrimination nor increased familiarity of stimulus colors. Finally, we replicated the principal effect of superior color working memory in an independent sample of synesthetes. Cumulatively, these results indicate that synesthetes exhibit enhanced dimension-specific visual working memory.

The current results significantly extend previous research on episodic memory advantages among synesthetes (Gross et al., 2011; Radvansky et al., 2011; Rothen & Meier, 2010; Rothen et al., 2012; Yaro & Ward, 2007) by showing that superior memory in this group is not restricted to long-term storage or retrieval and is already present in working memory. The results are notably consistent with those of Yaro and Ward (2007), who found that grapheme–color synesthetes exhibited greater color recognition memory than non-synesthetes, even for stimuli that do not elicit synesthetic color photisms (see also Rothen & Meier, 2010). Indeed, it is plausible that enhanced color working memory subserves superior color recognition memory in this population. Recently, Arnold et al. (2012) showed that grapheme–color synesthetes were more precise than controls at recalling the color and luminance of a colored circle using a modifiable color patch. Insofar as participants completed the recollection task only 500 ms after stimulus presentation, this result is indicative of superior color working memory among synesthetes and thereby bolsters the present results. One limitation of their study is that there were no time restrictions in the color recollection task and thus synesthetes may have outperformed controls by spending more time on the task. The present results, however, cannot be explained by this confound.

However, unlike in some episodic memory tasks (Gross et al., 2011; Radvansky et al., 2011; Yaro & Ward, 2007), synesthetes did not display superior working memory for inducer stimuli. We did observe a greater congruency effect (slower RTs for incongruent than congruent inducer graphemes) when participants were attending to grapheme colors. However, this effect was due to slower response latencies for incongruently-colored graphemes, plausibly the result of increased response conflict. Across experiments, synesthetes did not display a processing advantage for congruent graphemes relative to controls and the effect sizes for these effects were consistently near zero, with correspondingly low Bayes factors for the synesthetic Congruency effect in response accuracy, both of which support the null hypothesis (Dienes, 2011). These results are also partly consistent with those of Radvansky et al. (2011), who did not observe a specific advantage for congruently-colored words than achromatic words among synesthetes and also found that synesthetes’ advantage in word recall was not specific to congruent stimuli. Importantly, these results go against a dual-coding (Paivio, 1969, 1986) account of working memory in synesthesia (see also Yaro & Ward, 2007). At the same time, they are consistent with results showing that synesthetes do not reliably display superior immediate memory (Gross et al., 2011; Rothen & Meier, 2010) for inducer stimuli and do not reliably exhibit congruency effects in memory tasks (Rothen & Meier, 2009; Yaro & Ward, 2007). One possible explanation for the inconsistent observation of congruency effects on memory in synesthesia is that synesthetes may not consistently encode the grapheme and color in separate slave systems. For instance, both may be maintained in a phonological loop, and thus dual-coding benefits would not be expected. Alternatively, it may be that color photisms, which do not affect memory in the same way as sensory experiences (e.g., Arnold et al., 2012), do not confer an auxiliary coding advantage in working memory or short-term memory for graphemes in the same way as concurrent sensory experiences do (see also Mastroberardino et al., 2008; Rothen & Meier, 2010). Further research is needed to discriminate between these possibilities.

The observation that synesthetes do not have superior grapheme working memory is noteworthy in two other ways. First, this result strongly suggests that the observed differences in color working memory are not artifacts of differential motivation across groups. Synesthetes are cognizant of the fact that they are a special population and there is always an elevated risk that superior performance in this group can be attributed to increased motivation. Accordingly, insofar as greater motivation should lead to better performance across tasks, our finding that superior working memory among synesthetes is specific to color strongly discounts a differential motivation explanation (see also Gross et al., 2011; Radvansky et al., 2011). Second, the differing results across attended visual dimensions provide refined information regarding their putative neurocognitive locus. If synesthetes displayed enhanced domain-general working memory, this might suggest superior frontal modulation of fusiform gyrus and visual cortex in this population (Gazzaley & Nobre, 2012). Instead, the observed dissociation between color and grapheme working memory more strongly suggests a low-level mechanism related to enhanced color processing (Yaro & Ward, 2007). A further piece of evidence that isolates the locus of enhanced working memory in synesthetes to color processing and not domain-general working memory is that we did not observe Load × Group interactions on color working memory. That is, increased working memory load taxed performance to a relatively similar degree in the two groups. This suggests that synesthetes have superior baseline working memory and that increasing working memory load has a similarly deleterious effect on performance in synesthetes and non-synesthetes.

According to the enhanced processing account, color information is encoded more strongly in color synesthetes leading to higher fidelity color representations and, in turn, superior maintenance in working memory. This interpretation is consistent with multiple studies showing enhanced color (Arnold et al., 2012; Banissy et al., 2009; Yaro & Ward, 2007) and enhanced visual processing in the parvocellular pathway (Barnett et al., 2008) in color synesthetes. More broadly, given these documented differences between synesthetes and controls in visual processing, the current results provide further evidence for the proposal that brain regions responsible for processing and representing information are similarly responsible for the maintenance of that information in working memory (Jonides et al., 2005; Postle, 2006; Serences et al., 2009). Enhanced color working memory among color synesthetes may be subserved by hyperexcitability in primary visual cortex (Terhune, Tai, Cowey, Popescu, & Cohen Kadosh, 2011). Hyperexcitability may lead to the pooling of neurons in primary visual cortex tuned to stimulus color, which may amplify this feature or facilitate the reduction or exclusion of internal and external noise, respectively (Lu & Dosher, 2009), thereby producing a more stable representation. A corollary of this account is that synesthetes will display enhanced modality- or dimension-specific working memory pertaining to the modality of their concurrent (see also Simner, Mayo, & Spiller, 2009). For example, we would expect that mirror-touch synesthetes will display superior working memory for tactile information, but not for color information (see also Banissy et al., 2009), or that spatial-sequence synesthetes (Cohen Kadosh, Gertner, & Terhune, 2012) will display superior spatial working memory.

Enhanced color processing in synesthetes is likely to play a more fundamental role in the development and maintenance of synesthesia. Insofar as individual differences in working memory predict associative learning (Lewandowsky, 2011), enhanced color working memory may subserve learning of inducer–color pairs in early development, such as during the exposure to grapheme–color pairs (e.g., Witthoft & Winawer, 2013); indeed, synesthetes are better at learning novel symbol-color pairs than non-synesthetes (Rothen & Meier, 2010). Superior coding of colors may also be reflected in better color perceptual memory and thereby contribute to consolidation, and greater consistency, of extant inducer–color pairs (Yaro & Ward, 2007). However, a challenge for this account will be to determine the mechanisms underlying the specificity of synesthesia, that is, why an individual will develop grapheme–color, but not sound–color, associations, both of which are frequently reported by synesthetes (e.g., Niccolai, Jennes, Stoerig, & Van Leeuwen, 2012).

One non-competing alternative explanation of our results is that grapheme–color synesthetes display broader, enhanced processing in the parvocellular visual pathway (Barnett et al., 2008; Rothen et al., 2012), which enables processing of color and high contrast stimuli (e.g., Brown, 2009). This hypothesis more readily explains synesthetes’ superior memory for achromatic visual stimuli that do not elicit color photisms (Rothen & Meier, 2010) than dual-coding and enhanced color processing accounts. However, a parvocellular-specific processing advantage should still have produced an advantage in grapheme working memory in synesthetes, which we did not observe to a great degree.

In summary, grapheme–color synesthetes displayed greater color working memory than non-synesthetes, whereas the two groups did not differ in grapheme working memory. Such superior color working memory in synesthetes is not attributable to superior color discrimination, the online experience of synesthesia, or color familiarity. Cumulatively, these results demonstrate superior dimension-specific working memory in grapheme–color synesthesia, which may be subserved by enhanced color processing in this population. Beyond synesthesia, these results provide a clear demonstration of how visual working memory can be constrained in a dimension-specific manner and supplies further evidence for a close relationship between sensory processing and the maintenance of sensory information in working memory.

## Figures and Tables

**Table 1:** Descriptive statistics [*M* and (*SD*)] for *n*-back conditions in experiment 1, 2, and 3 in controls and grapheme–color synesthetes*.*

Variable	Experiment 1				Experiment 2				Experiment 3			
	Controls		Synesthetes		Controls		Synesthetes		Controls		Synesthetes	
	ER	RT	ER	RT	ER	RT	ER	RT	ER	RT	ER	RT
*Inducer graphemes*												
2-Back congruent foils	.19 (.10)	604 (111)	.13 (.06)	646 (125)	.16 (.08)	697 (106)	.16 (.10)	733 (193)				
2-Back congruent targets	.18 (.12)	581 (121)	.10 (.07)	589 (104)	.18 (.10)	698 (139)	.16 (.12)	685 (162)				
2-Back incongruent foils	.19 (.08)	606 (107)	.15 (.09)	654 (124)	.20 (.10)	712 (100)	.16 (.13)	732 (200)				
2-Back incongruent targets	.22 (.16)	547 (120)	.13 (.08)	586 (128)	.20 (.10)	673 (131)	.20 (.18)	677 (181)				
3-Back congruent foils	.29 (.12)	603 (142)	.22 (.15)	705 (150)	.31 (.13)	815 (148)	.22 (.14)	756 (213)				
3-Back congruent targets	.39 (.14)	552 (103)	.28 (.16)	635 (141)	.32 (.14)	707 (135)	.29 (.18)	721 (207)				
3-Back incongruent foils	.28 (.14)	593 (114)	.22 (.15)	699 (148)	.30 (.10)	781 (173)	.24 (.13)	747 (198)				
3-Back incongruent targets	.43 (.18)	597 (148)	.29 (.20)	678 (140)	.30 (.09)	738 (214)	.26 (.22)	722 (184)				

*Non-inducer graphemes*												
2-Back foils	.20 (.08)	593 (107)	.14(.ll)	642 (125)	.21 (.18)	710 (116)	.15 (.09)	737 (201)	.18 (.07)	697 (140)	.10 (.06)	501 (78)
2-Back targets	.18 (.13)	552 (103)	.08 (.05)	569 (119)	.23 (.11)	650 (103)	.19 (.09)	683 (203)	.15 (.14)	666 (103)	.13 (.11)	485 (93)
3-Back foils	.26 (.13)	587 (113)	.19 (.12)	697 (175)	.30 (.13)	761 (185)	.22 (.13)	754 (189)	.30 (.08)	771 (172)	.20 (.08)	561 (127)
3-Back targets	.42 (.23)	591 (138)	.25 (.15)	639 (170)	.43 (.16)	732 (209)	.43 (.18)	766 (248)	.27 (.13)	748 (157)	.28 (.13)	522 (107)

## Footnotes

1 Superior low-level visual processing in synesthetes (Barnett et al., 2008) may also entail superior working memory for graphemes and thus would provide an alternative explanation for superior grapheme working memory to the dual-coding hypothesis, although unlike the latter, such an account would not predict Congruency effects.

2 Yaro and Ward (2007) do not report descriptive statistics and thus this effect size was estimated on the basis of the respective figure.

3 Further exploratory ANOVAs revealed that whilst controls and synesthetes did not differ on congruent trials, *F* < 1.6, synesthetes were suggestively slower than controls on incongruent trials, *F*(1, 30) = 3.13, *MSE *= 12,555, *p* = .087, $\eta_{p}^{2}=.09$ (95% CIs: .00, .31). Insofar as there was a Congruency effect on ERs and differential congruency effects on RTs, we repeated the ANOVA using efficiency (RT/(1 − ER)) as the dependent variable. There were no Congruency or Group effects, *F*s < 2.9, nor a Congruency × Group interaction, *F *< 1.1.

4 It could be objected that because digit-color consistency was only verified in a subset of our synesthetes, the remaining synesthetes might not exhibit robust congruency effects and, in turn, diminish the magnitude of Congruency effects in this group. We investigated this possibility by repeating the analyses with these two subgroups comprising the two levels of a between-groups independent variable; there were no differences between the two groups in the magnitude of Congruency effects in response accuracy or latency, nor any of the diffusion parameters, *F*s < 3.6.

5 As in Experiment 1, we investigated whether Congruency effects varied as a function of whether synesthetes’ consistency had been verified or not (cf. Footnote 4). Again, there were no differences between these two groups, *F*s < 3.0.
